# Zeolitic Imidazole Framework (ZIF)–Sponge Composite for Highly Efficient U(VI) Elimination

**DOI:** 10.3390/molecules29020408

**Published:** 2024-01-15

**Authors:** Shengxia Duan, Xinshu Long, Jian Liu, Xiaomin Jin, Guihong Zhao, Jiaxing Li, Zaidao Liu

**Affiliations:** 1 Department of Chemistry and Engineering, Heze University, Heze 274500, China; duanshengxia@hezeu.edu.cn (S.D.); longxinshu@sina.com (X.L.); 17661369763@163.com (X.J.); 2 CAS Key Laboratory of Photovoltaic and Energy Conservation Materials, Institute of Plasma Physics, Chinese Academy of Sciences, Hefei 230031, China; lijx@ipp.ac.cn; 3 College of Agriculture and Bioengineering, Heze University, Heze 274000, China; 4 China National Nuclear Corporation Shaoguan JinYuan Uranium Co., Ltd., Shaoguan 512000, China; f15964152031@163.com

**Keywords:** ZIF-67, PU sponge, U(VI) elimination, mechanism, practical application

## Abstract

Herein, a zeolitic imidazole framework (ZIF-67) composite was prepared by a rapid, simple and inexpensive situ hybridization technique applying polyurethane sponge (PU) as support, which was designated as ZIF-67-PU. The ZIF-67 nanoparticle was successfully supported on the surface of sponge skeletons mainly through electrostatic attraction as well as probable π−π stacking interactions with PAM modification of the sponge. The resultant ZIF-67-PU exhibited a remarkably enhanced U(VI) elimination capacity of 150.86 mg∙g^−1^ on the basis of the Langmuir isotherm model, in comparison to pristine sponge. Additionally, the mechanism for U(VI) elimination was mainly achieved through the complex reaction between C–N(H)/–OH groups in ZIF-67 and U(VI), based on XPS investigations. ZIF-67-PU represents a simple, feasible and low-cost disposal option for preparing ZIF-coated sponges of any shape that can enhance the U(VI) elimination capacity. Furthermore, this approach can be widely applied to the preparation of various kinds of MOF-sponges through this situ hybridization technique.

## 1. Introduction

The release of long-lived radionuclides, which mainly originated from civilian and military nuclear programs [1,2], reflects mounting concern that it could cause harmful effects to human health and environmental pollution management [3]. Among all these long-lived radionuclides, uranium(VI), as a typical actinide element, has both radioactivity and chemical toxicity, which could cause irreparable damage to human biological functions, such as severe liver damage, urinary system ailments, DNA injury, etc. [4,5,6]. In addition, an interim guideline value of U(VI) specified by the World Health Organization is only 30 μg∙L^−1^ in drinking water. Hence, it is of imperative significance to eliminate and recover U(VI) from aqueous solution. Until now, a series of methods, such as ion-exchange [7,8], chemical precipitation [9], electrochemical extraction [10,11], and adsorption [12,13,14,15], have been employed for the elimination of U(VI) from solutions. Among them, adsorption technology is of particular interest to eliminate U(VI) from wastewater because of its large number of positives, such as low cost, elevated removal efficiency and easy operation on a large scale [16]. Therefore, investigating advanced adsorbents with great elimination capacities and rates is a definitive direction for U(VI) removal from aqueous solution.

In general, absorbent materials, which contain a large number of functional groups in their outer structure, can realize efficient U(VI) elimination from wastewater [17]. The commendable adsorbent materials for U(VI) elimination from wastewater should possess the following characteristics: (i) facile synthesis and large-scale production, (ii) environmentally friendly and low cost, (iii) high stability of mechanical structure to radiation, (iv) fast elimination rate and high capacities. As a new kind of porous material with a tunable pore size, large specific surface area and a rich variety of functional groups, metal–organic frameworks (MOFs) can inherit these all these features. And various MOFs materials have been applied for U(VI) elimination from wastewater because of their large numbers of reactive sites and mechanical robustness against wear and tear. For instance, Zhao and his co-workers successfully combined Fe_3_O_4_, HKUST-1, and GO to obtain a magnetic sphere hybrid ternary composite (Fe_3_O_4_@HKUST-1/GO) via the hydrothermal method, which possessed excellent properties of a carbonaceous graphene surface with high porosity and adjustability of MOFs and magnetic separation. And the Fe_3_O_4_@HKUST-1/GO exhibited a good adsorption capacity of 268.82 mg∙g^−1^ towards U(VI) at the initial solution pH value of 4.0 and T = 318 K [18]. Moreover, amidoxime was reported as the most promising group for U(VI) elimination because of its relatively high affinity and selectivity toward UO_2_^2+^. Based on this finding, Wu et al. combined UiO-66 with acrylonitrile to prepare amidoxime-appended UiO-66 (UiO-66-AO) for the efficient and rapid removal of U(VI) from aqueous solution and its maximum adsorption capacity reached 232.8 mg∙g^−1^ at pH 5.0 and 328 K based on the Langmuir isotherm [19]. Furthermore, doping metal elements with similar valence states and ionic radius can endow MOFs with extra adsorption sites on the basis of maintaining the MOF framework. Hence, Chen et al. successfully prepared Fe-doped MOF material (Fe-ZIF-8-300), and its maximum adsorption capacity reached 343.5 mg∙g^−1^ at pH 4.5 and 298.15 K [20]. Moreover, phosphate-derived materials, especially hydroxyapatite (HAP), have attracted widespread attention in U(VI) adsorption, since they contain abundant binding groups, such as phosphate (PO_4_^3−^) and hydroxy groups (Ca-OH), which can complex and mineralize with U(VI). Based on this consideration, Guo and his co-workers synthesized an HAP-modified ZIF-67 composite (HAP/ZIF-67) and its maximum adsorption capacity reached 453.1 mg∙g^−1^ at pH 5 and 298 K [21]. Additionally, UiO-66-NH_2_ has been shown to efficiently eliminate U(VI) from aqueous solution [22].

However, these nanoscale MOF materials tend to clump and agglomerate rapidly in aqueous solution, which could result in blocking the active sites, retarding the rate of U(VI) elimination and leading to difficulties in its separation and recovery from wastewater. Therefore, the complex and costly post-separation of these nanoscale particles from wastewater should be solved to promote the practical application of MOF-based adsorbents in the industrial process. The immobilization of nanoscale particles on suitable substrates is the most common approach to realize the instant separation from wastewater. Nowadays, sponge, which has a skeleton of a three dimensional (3D) porous structure, has been widely applied as a substrate for the immobilization of nanoscale materials [23]. And the sponge substrate acts as a 3D support for coating to avoid the agglomeration of nanoscale particles, meanwhile the bulk structure facilitates the recycling of the used adsorbents.

**Scheme 1:**
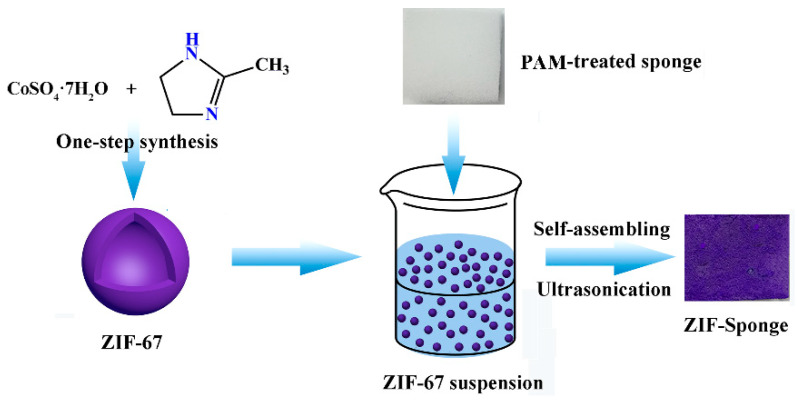
Schematic fabrication of ZIF-67-PU.

Herein, an in situ hybridization technique was proposed to rapidly and simply prepare the composites consisting of both MOF materials and sponge. To demonstrate this approach, ZIF-67 was chosen as the representative MOF because of its facile and mild synthesis procedure [24,25]. Moreover, the BET surface area of the ZIF-67 could be larger than 1000 m^2^∙g^−1^, which would be beneficial to affording much more active sites to capture the contaminant from aqueous solution. Furthermore, the commercially available polyurethane sponge (PU) was chosen as the substrate material for supporting ZIF-67 to compare their effects on the elimination performance of U(VI). In addition, polyacrylamide (PAM) was also applied to functionalize sponges in order to accelerate the ZIF-loading on sponges. The schematic illustration of ZIF-67-PU is shown in Scheme 1.

## 2. Results and Discussion

### 2.1. Properties of the Synthesized Samples

**Figure 1:**
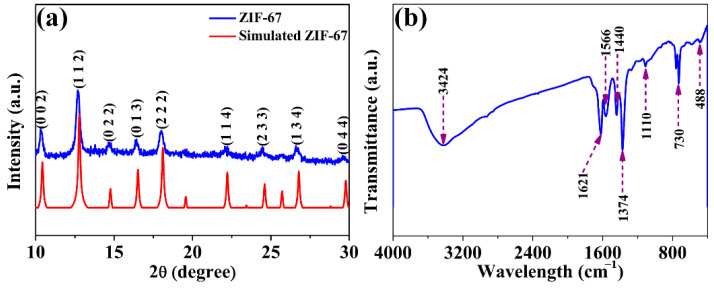
XRD pattern (**a**) and FT-IR spectra (**b**) of obtained ZIF-67 material.

The crystalline phase and purity of the samples were confirmed by XRD patterns. The obtained XRD spectrum was in excellent agreement with the simulated structure [26,27], as shown in Figure 1a. ZIF-67 has many diffraction peaks, especially in the 2θ range of 5–30°, and distinguishable diffraction peaks appear at 2θ = 10.3°, 12.7°, 14.7°, 16.4°, 18.0°, 22.1°, 24.4°, 26.6° and 29.6° corresponding to the crystal planes (0 0 2), (1 1 2), (0 2 2), (0 1 3), (2 2 2), (1 1 4), (2 3 3), (1 3 4) and (0 4 4), respectively. In addition, the XRD pattern of ZIF-67 presented no impurity peaks, indicating that the ZIF-67 material was successfully synthesized and had high purity. Furthermore, the XRD patterns of ZIF-67 after U(VI) adsorption are shown in Figure S1. It can be seen that the shape and peak positions of the ZIF-67 after U(VI) adsorption are almost unchanged, suggesting the stability of ZIF-67 under solution pH 5.5.

FT-IR analysis was carried out to characterize the surface organic functional groups of the obtained material. The absorption peak with a broad band situated at 3424 cm^−1^ belongs to the stretching vibrations of –OH, originating from surface hydroxyl groups and coordinated H_2_O molecules. According to a previous report [28], the FT-IR spectrum of the N–H∙∙∙N hydrogen bond of MeimiH can be located at 3400 to 2200 cm^−1^, exhibiting a strong and broad band. And the band at 1846 cm^−1^ can be ascribed to N–H stretching vibration [28]. However, there is no appearance of the strong and broad bands in the obtained ZIF-67 nanoparticles, suggesting the N–H groups of MeimiH deprotonated upon coordinating with Co(II). The peak at 1566 cm^−1^ results from the C=N stretching vibration on the imidazole ring. The appearance of peaks at 1440 and 1374 cm^−1^ were attributed to the tensile vibration of the entire ring. The peaks that appeared at 1110 cm^−1^ and 730 cm^−1^ corresponded to in-plane and out-of-plane bending of the imidazole ring, respectively [29,30]. Furthermore, a new adsorption band appeared at 488 cm^−1^, indicating the successful complexation of metal ions with ligands. Additionally, the spectra of ZIF-67 obtained in this study were in line with the previously reported values [31], further confirming the successful coordination of MeimiH and Co(II) in the framework.

**Figure 2:**
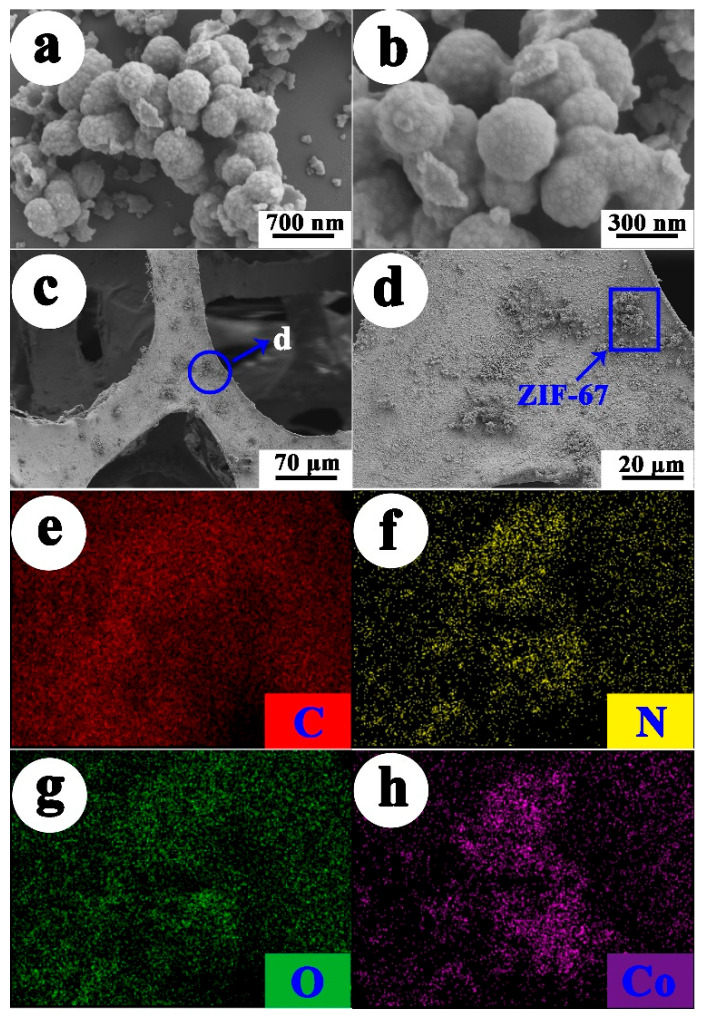
SEM images of the ZIF-67 and ZIF-67-PU (**a**) ZIF-67, (**b**) magnified image of ZIF-67, (**c**) ZIF-67-PU, (**d**) magnified image of ZIF-67-PU, (**e**–**h**) elemental mapping images of ZIF-67-PU.

The morphologies and structures of the obtained ZIF-67 and ZIF-67-PU were characterized by SEM as shown in Figure 2. Figure 2a,b displays the SEM image and magnified image of ZIF-67, respectively. It can be clearly seen that ZIF-67 particles exhibited hollow nano-sphere morphology with a diameter of about 600 nm. This special hollow structure is beneficial in enhancing its BET surface area and further supply more active sites for U(VI) elimination. Figure 2c,d exhibit SEM images of ZIF-67-PU. It can be clearly observed that ZIF-67 nanoparticles were successfully supported on the surface of PU sponge mainly through electrostatic attraction as well as probable π−π stacking interactions with PAM modification of the sponge, further certified by elemental mapping images (Figure 2e–h) and EDS characterization as shown in Figures S2 and S3.

**Figure 3:**
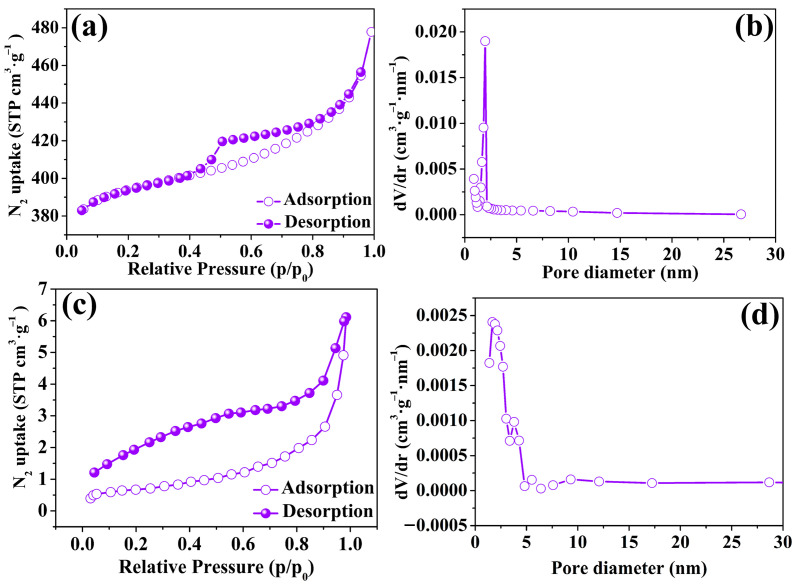
(**a**,**c**) N_2_ adsorption–desorption isotherms of ZIF-67 nanoparticles and ZIF-67-PU, respectively; (**b**,**d**) pore size distribution plots of ZIF-67 nanoparticles and ZIF-67-PU calculated using the BJH method.

The pore structures of ZIF-67 nanoparticles and ZIF-67-PU were characterized by the N_2_ adsorption–desorption method. The N_2_ adsorption isotherm of ZIF-67 exhibited a type IV isotherm with a small H1 hysteresis. When the relative pressure was less than 0.01, the adsorption curve rapidly rose, thereby suggesting the occurrence of adsorption in the micropores. Meanwhile, the hysteresis loop at high pressures (0.4–1.0) in the isothermal curve indicated the presence of mesopores (Figure 3a). Moreover, the presence of both micropores and mesopores can be ascribed to the heterogeneity of the shape of the pores. And its BET specific surface area was as high as 1190.0 m^2^∙g^−1^, which was also in line with the previously reported values of ZIF-67-based composites [23,26,27]. Furthermore, the BET area of composite ZIF-67-PU was 3.0 m^2^∙g^−1^ (Figure 3b), which experienced a threefold increase in comparison to raw PU sponge (1.0 m^2^∙g^−1^, shown in Figure S3). Moreover, Figure 3c,d showed the pore size distribution of ZIF-67 and ZIF-67-PU calculated with the BJH desorption method, respectively. The ZIF-67 nanoparticles clearly demonstrated an average pore diameter of 2.74 nm. The average pore diameter of raw PU was 1.945 nm with a pore volume of 0.017 cm^3^∙g^−1^, while the average pore diameter of ZIF-67-PU was only 1.673 nm with a pore volume of 0.01 cm^3^∙g^−1^. It can be inferred that the deposition of ZIF-67 nanoparticles onto PU could compress its volume, leading to a decrease in its pore volume and further decreasing its average pore diameter. Furthermore, according to the aforementioned results, they demonstrate abundant functional groups, a high surface area, and mesoporous size distribution, meaning that the samples may become one of the most efficient materials for U(VI) elimination. Additionally, the corresponding parameters of pore characteristics are illustrated in Table 1. And the N_2_ adsorption/desorption isotherms of ZIF-67 and ZIF-67-PU after U(VI) adsorption demonstrated that the original pore structures of the obtained adsorbents were retained well (Figure S4). Above all, the adsorbents obtained in this study have good structure stability.

### 2.2. Contact Time Effect and Kinetics Study

**Figure 4:**
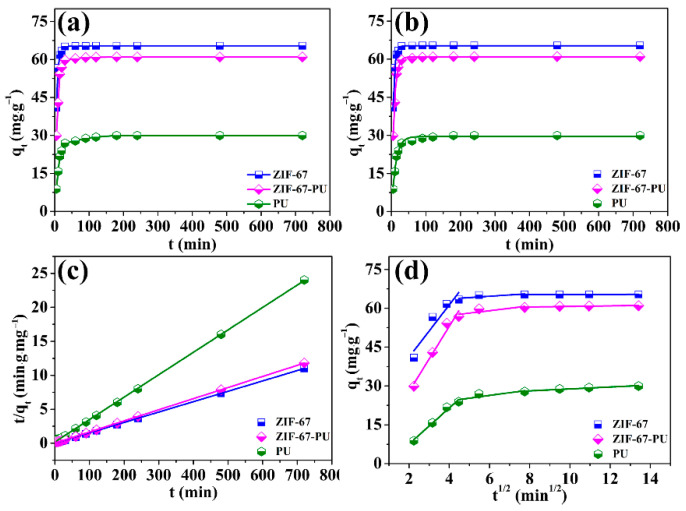
(**a**) Effect of contact time, (**b**) pseudo-first-order model, (**c**) pseudo-second-order mode and (**d**) intra-particle diffusion model plot for the elimination of U(VI) onto ZIF-67, ZIF-67-PU, and PU, pH = 5.0 ± 0.1, *C_U_*_(*VI*)(*initial*)_ = 33 mg·L^−1^, *m/V* = 0.5 g·L^−1^.

To compare the elimination efficiency between ZIF-67 and ZIF-67-PU, the impact of operation time on U(VI) elimination by the obtained samples (i.e., ZIF-67, PU and ZIF-67-PU) was experimentally studied. As can be seen from Figure 4, all the obtained adsorbents exhibited a relatively fast elimination rate at the beginning of the contact time and then gradually reached the adsorption equilibrium. The amount of absorbed U(VI) onto ZIF-67-PU was 63.07 mg∙g^−1^, which was about 2 times higher than that of PU (30.23 mg∙g^−1^). The ZIF-67 loaded onto sponge can greatly improve the elimination of U(VI), which can be ascribed to the increased active sites from ZIF-67. The dynamic processes were further analyzed using pseudo-first-order models, pseudo-second-order models, and intra-particle diffusion models to deepen our understanding of the elimination process [32,33,34]. The detailed descriptions of the above three kinetic models are summarized in Table S1. And the fitting results are shown in Figure 4b–d and Table 2. It can be seen that the adsorption kinetics of U(VI) onto the obtained adsorbents were fitted better by the pseudo-second-order model than the pseudo-first-order model due to its higher correlation coefficient (*R*^2^ > 0.999) and closer calculated value (*q_e_*_,*cal*_) to the experimental value (*q_e_*_,*exp*_), further corroborating the dominant chemisorption/surface complexation mechanism for U(VI) adsorption onto the obtained ZIF-sponges.

Figure 4d described the U(VI) elimination behavior on the ZIF-67-PU surface edges, obtained by fitting the kinetic data using the intra-particle diffusion model. It can be seen that the no curves crossed the origin, suggesting a multistep elimination process. Initially, the adsorption process displayed a faster elimination rate. And this phenomenon may result from the high concentration of U(VI) and large number of active sites, which was favorable for the mass transfer process. Afterwards, the elimination rate decreased, due to the limited number of active sites and reduction in the U(VI) concentration. Finally, the intercepts of the above fitted curves approached zero, suggesting that the intra-particle diffusion may be involved in this elimination process [35]. And according to the analysis of the above kinetics models, the U(VI) elimination process onto ZIF-67-PU can be described as follows: (i) U(VI) ions reached the surface of the adsorbent by liquid film diffusion and (ii) U(VI) ions realized their diffusion into the holes of mesoporous materials to acquire their complexation reaction with functional groups on ZIF-67-PU surfaces.

### 2.3. pH and Ionic Strength Effect

**Figure 5:**
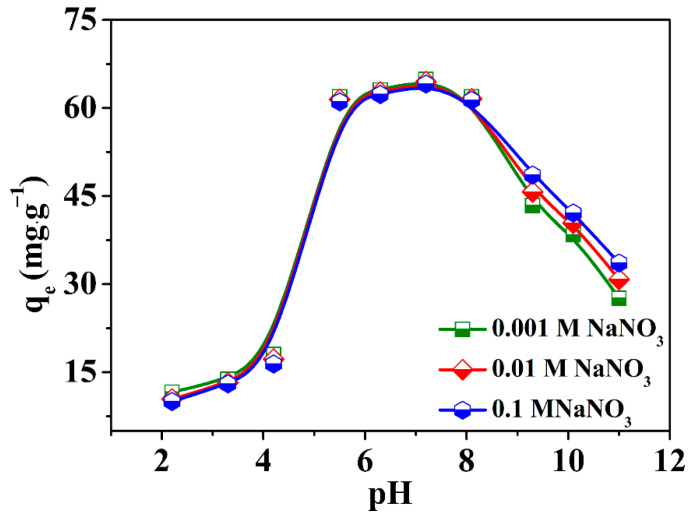
Effect of pH and the ionic strength on the adsorption of U(VI) onto the obtained ZIF-67-PU, *T* = 298 K, *C_U_*_(*VI*)*initial*_ = 33 mg·L^−1^, *m*/*V* = 0.5 g·L^−1^.

Since the solution pH value could have a large influence on the adsorption process, the adsorption experiments of U(VI) concerning the pH effect were carried out in the pH scale of 2 to 12. As shown in Figure 5, the U(VI) elimination performance increased significantly from 2 to 5, and then remained relatively constant in the pH scale of 5−7, while the U(VI) elimination performance suffered a sharp decrease from 7 to 12. From this phenomenon, it can be inferred that the ZIF-67-PU could be denoted as an effective adsorbent in the U(VI) elimination procedure carried out under neutral and weak alkali conditions.

The dominant U(VI) species were UO_2_^2+^ and UO_2_OH^+^ when the acidity of the solution was no more than 5, as shown in Figure S5 and Table S2. The positive and negative charge of ZIF-67-PU were found at pH < 6.7 and pH > 6.7, respectively, given that the pH_pzc_ of raw ZIF-67 was 6.7 based on the zeta potential test (Figure S6). Hence, the poor U(VI) elimination performance in lower environmental pH values can be ascribed to the electrostatic repulsion between uranium cations (UO_2_^2+^, UO_2_OH^+^) and positively charged surface of ZIF-67-PU. The great U(VI) elimination performance of ZIF-67-PU in the pH scale of 5.0–8.0 may be a result of the strong affinity between the adsorbent and uranium multi-nuclear hydroxides. And the decreased adsorption of U(VI) at pH > 7.0 could also be explained by the static repulsion between negatively charged ZIF-67-PU and negatively charged multiple hydroxyl radicals (e.g., UO_2_(OH)_4_^2−^). Therefore, the following elimination experiments were carried out under pH 5.5, making sure that no precipitation or hydrolysis of U(VI) occurred in the adsorption experiments to achieve a better elimination performance.

The impacts of ion strength on the process of U(VI) elimination by ZIF-67-PU were measured using different concentrations of NaNO_3_ over a wide range of solution pH values. From Figure 5, it can be seen that the ionic strength had a low impact on the U(VI) elimination process carried out by ZIF-67-PU when the pH of the solution was less than 8, which revealed that the interaction mechanism was primarily inner-sphere surface complexation [35]. However, an increase in ion strength was beneficial to improving the adsorption performance of U(VI) onto ZIF-67-PU when the pH of the solution exceeded 8, suggesting an out-sphere surface complexation mechanism. Based on previous studies [36,37,38], the concentration of the background liquid ions could substantially impact the thickness of the diffused electric double coating as well as the potential of the substances, impacting the binding of U(VI) to different materials. Generally speaking, the inner-sphere complexation interaction was more insusceptible to the alterations of the background ions in comparison with the outer-sphere complexation reaction. Although ion strength has a positive effect on U(VI) adsorption at higher pH values, there was no significant difference in the total adsorption efficiency of U(VI), indicating that U(VI) elimination onto ZIF-67-PU was mainly realized through the inner-sphere complexation between U(VI) and uncoordinated C–N(H)/–OH groups on the ZIF-67 surface rather than outer-sphere surface complexation, which was further certified by XPS analysis. Based on the above discussion, ZIF-67-PU composites are anticipated to be effective for removing U(VI) from water environments even at high salt concentrations.

### 2.4. Adsorption Isotherms

**Figure 6:**
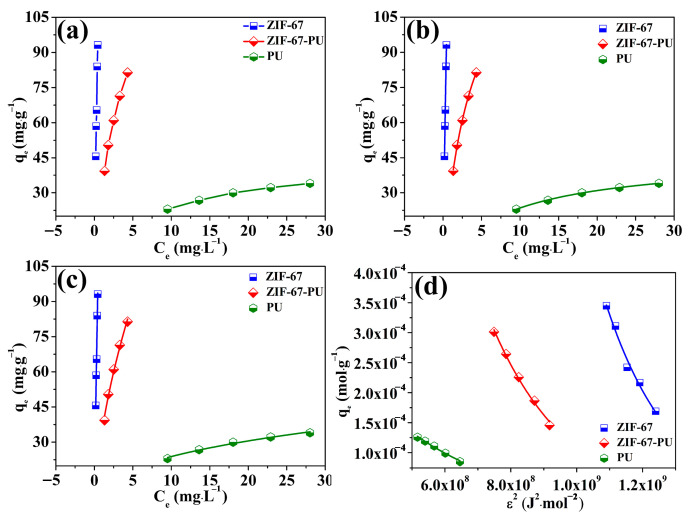
Isotherm models for U(VI) elimination on the obtained adsorbents (**a**), Langmuir (**b**), Freundlich (**c**), D-R model (**d**), (pH = 5.5 ± 0.1).

To achieve a better understanding of the U(VI) elimination behavior onto ZIF-67-PU, its adsorption isotherm tests were conducted by changing the initial concentrations of U(VI) in the range of 21–45 mg∙L^−1^ at 298 K. The results are shown in Figure 6a. It can be clearly seen that the adsorption capacity of U(VI) significantly enhanced with increasing initial U(VI) concentrations and the enhanced extents of U(VI) adsorption remarkably decreased at high initial U(VI) concentrations. The adsorptive experiment data were ulteriorly simulated by Langmuir, Freundlich and Dubinin–Radushkevich (D-R) models [39,40,41]. The Langmuir isotherm model indicates that adsorption occurs on the surface of the homogeneous adsorbent and that the distribution of all the adsorption sites is uniform on the adsorbent surface. However, the Freundlich Isotherm model indicates an inhomogeneous surface system, suggesting that the active sites are different on the adsorbent surface, which will further lead to differences in adsorption energies as well as unevenly distributed surface heat. And this result would eventually promote the arrival of the adsorbate to the inhomogeneous adsorbent surface from the liquid environment [42]. The above two isotherm models can be illustrated as follows:

Langmuir isotherm model:

(1) $$q_{e}=\frac{Q_{\max}bC_{e}}{1+bC_{e}}$$

Freundlich isotherm model:

(2) $$q_{e}=K_{f}C_{e}^{1/n}$$

In the above models, *C_e_* presents the concentration (mg∙L^−1^) of U(VI) under equilibrium conditions, *Q_max_* presents the maximum adsorption capacity (mg∙g^−1^), *q_e_* presents the adsorption amount (mg∙g^−1^) when adsorption reached equilibrium on ZIF-67-PU and *b* (L·g^−1^) in Equation (1) presents the Langmuir adsorption constant. *K_F_* presents the Freundlich constant (L∙mg^−1^). 1/*n* presents the degree of the adsorption-dependent equilibrium concentration.

The fitting results are illustrated in Figure 6b,c and Table 3, which indicated that the Langmuir isotherm model was more applicable in describing the elimination of U(VI) onto ZIF-67-PU because of its higher correlation coefficient, compared to that obtained from the Freundlich isotherm model. The maximum elimination capacity of U(VI) onto ZIF-67-PU based on the Langmuir isotherm model was 150.86 mg∙g^−1^, although the maximum elimination capacity of PU was only 45.16 mg∙g^−1^, which clearly indicated that the greatly enhanced U(VI) elimination capacity onto the PU sponge can be ascribed to the successful loading of ZIF-67 nanoparticles on their surfaces. Table 4 compared the maximum elimination capacity of U(VI) onto other adsorbents reported in previous studies [20,43,44,45,46,47,48,49,50,51,52,53,54,55]. It could be seen that ZIF-67-PU possessed a lower elimination capacity in comparison to other MOF adsorbents, i.e., HAP/ZIF-67 [22], BZCA-ZIF-8 [52], Fe_3_O_4_@ZIF-8 [53], and PAN@ZIF-8 [54], which can be ascribed to the limited amounts of ZIF-67 nanoparticles on the PU surface, since the functional groups of MOF materials played a dominant role in improving the elimination capacity. Moreover, the surface area also has an important influence on adsorption performance. The BET surface area of ZIF-67-PU is only 2.57 m^2^·g^−1^, much lower than that of Fe-ZIF-8-300(PO_4_) (755.25 m^2^·g^−1^ and 1069.42 m^2^·g^−1^) [20], HAP/ZIF-67 (945.4 m^2^·g^−1^) [22], BZCA-ZIF-8 (220.45 m^2^·g^−1^) [52] and so on, which is responsible for its lower adsorption capacity for U(VI). Although ZIF-67-PU is insufficient in its elimination capacity in comparison to other efficient MOF adsorbents, the ZIF-sponge displayed remarkable U(VI) elimination performance compared to other adsorbents such as biochar (2.12 mg∙g^−1^) [43], silicate/diatomite (31.54 mg∙g^−1^) [44], sulfonated graphene oxide (45.05 mg∙g^−1^) [45], ZIF-90 (77.6 mg∙g^−1^) and ZIF-90-SA (85.1 mg∙g^−1^) [46]. This phenomenon clearly reflects that ZIF-67-PU could be effectively used in managing uranium-bearing wastewater.

The other isotherm model, Dubinin–Radushkevich (D-R), was also applied to analyze whether the elimination process was physical or chemical. The equation was given as follows:

(3) $$q_{e}=Q_{\max}\exp(-\beta \varepsilon^{2})$$

(4) $$\varepsilon =RT\ln(1+\frac{1}{C_{e}})$$

(5) $$E={\left(2\beta \right)}^{-1/2}$$

in which *β* in Equation (3) is the representative of the activity coefficient (mol^2^·kJ^−2^) and *ε* is the representative of Polanyi’s adsorption potential. *E* (kJ·mol^−1^) represents the adsorption energy, which is applied as one of the basic parameters to certify whether this process of U(VI) elimination is a chemical-driven or physical-driven process. On the basis of previous reports [56,57], the *E* value of 8–16 kJ·mol^−1^ suggests a chemical-driven elimination procedure, while the *E* value is lower than 8 kJ·mol^−1^, demonstrating a physical-driven elimination procedure. As shown in Figure 6d and Table 3, the result values of *E* obtained in this elimination process vary from around 8 kJ·mol^−1^ to 16 kJ·mol^−1^, suggesting a chemical-driven U(VI) elimination procedure by ZIF-67-PU. Moreover, this result also indicates that the deposition of ZIF-67 onto PU sponge brought no change in its elimination nature behavior.

Furthermore, the concentrations of Co released during the adsorption process under solution pH 5.5 at room temperature were tested to investigate the possible cobalt release during the adsorption process. The data are illustrated in Table S3. It can be seen that the concentration of Co released experienced an increase with the increase in the initial concentration of U(VI). This phenomenon can be ascribed to the competition of U(VI) and Co(II) to coordinate with nitrogen functional groups. Although some Co(II) ions were released during the adsorption process, it still can be ignored compared to the previous adsorbent concentration of *m/V* = 0.5 g·L^−1^, further confirming the relatively stable structure of ZIF-67.

**Figure 7:**
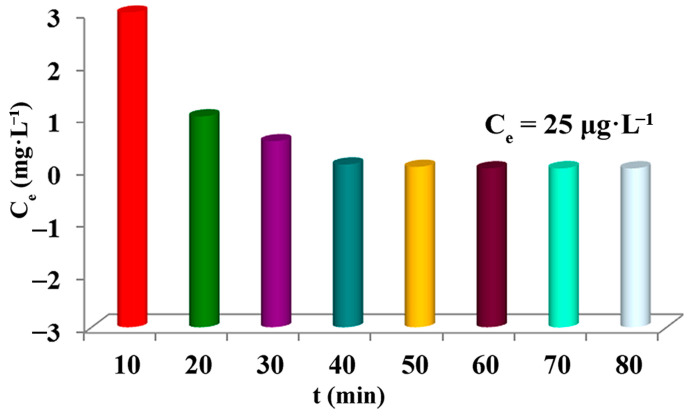
Adsorption efficiency of U(VI) removal by ZIF-67-PU.

In order to further realize the practical application of ZIF-67-PU, a large piece of PU deposited with ZIF-67 nanoparticles was brought into contact with U(VI)-containing solution with 800 mL of 9 mg·L^−1^. As shown in Figure 7, the vast majority of U(VI) can be removed by ZIF-67-PU within 60 min, reaching the uranium-bearing wastewater draining standard of 30 μg·L^−1^. This result made ZIF-loading sponges possible in practical application. Additionally, the equilibrium time of this experiment was significantly shorter than that of the kinetics study discussed above, which could be understood by the lower concentration of uranium-bearing wastewater and more abundant active binding sites.

### 2.5. Adsorption Mechanism

**Figure 8:**
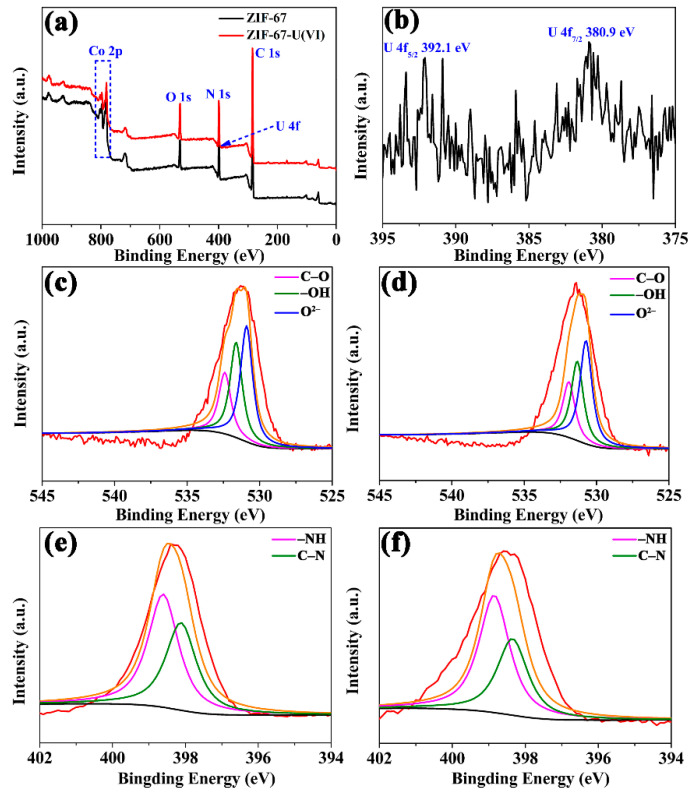
(**a**) XPS survey spectra of ZIF-67 and ZIF-67-U(VI), (**b**) XPS spectra of U(VI), (**c**,**e**) high-resolution XPS spectra of ZIF-67 for both O 1s and N 1s before U(VI) adsorption; (**d**,**f**) high-resolution XPS spectra of ZIF-67 for both O 1s and N 1s U(VI) immobilization (red line: raw intensity curves; orange line: fitted intensity curves).

XPS technology has been widely employed as a convenient and effective tool for the investigation of adsorption mechanisms. Figure 8 illustrates the full scan XPS spectra of ZIF-67 and ZIF-67-U(VI), displaying the dominant elements of Co, O, N and C in ZIF-67. Moreover, double U4f peaks (392.1 eV for U 4f_5/2_ and 381.2 eV for U 4f_7/2_) were observed in the full-scale XPS spectra of ZIF-67-U(VI) after elimination, further confirming the successful binding of U(VI) ions onto the ZIF-67 surface. Furthermore, the high-resolution spectra of N 1s and O 1s were discussed to obtain further insight into the key role of nitrogen and oxygen functional groups for U(VI) elimination. As shown in Figure 8c, O 1s can be deconvolved into three peaks, 532.40 eV, 531.62 eV and 530.90 eV, which can be attributed to C–O single bonds, –OH groups and O^2−^ ions, respectively. After U(VI) adsorption (Figure 8d), the binding energy of O atoms shifted to lower values of 531.91 eV, 531.33 eV and 530.73 eV, indicating that the surface complexation to U(VI) by oxygen-containing groups was not the only elimination mechanism. And the O atoms of ZIF-67 tended to chelate with U(VI) via covalent bonds, decreasing the binding energy of these O atoms. The N 1s spectra in Figure 8e before U(VI) adsorption can be fitted to two peaks of 398.61 eV and 398.12 eV, corresponding to –NH and C–N, respectively. After the U(VI) capture, the two peaks of N 1s shifted to higher binding energies of 398.85 eV and 398.35 eV (Figure 8f), suggesting that U(VI) ions could strongly interact with –NH and C–N groups. Therefore, it can be concluded that the U(VI) elimination mechanism can be attributed to the complexation interaction between U(VI) and nitrogen- and oxygen-containing functional groups on the surface of ZIF-67-PU.

## 3. Experimental Section

### 3.1. Materials and Reagents

Commercial polyurethane sponge was purchased from Langsheng sponge products Sales Co., Ltd. (Shanghai, China). Cobalt Sulfate (CoSO_4_·7H_2_O). Methanol and 2-methylimidazole (MeimiH) were commercially available from Sinopharm Chemical Reagent Co., Ltd. (Shanghai, China). The reserve U(VI) solution was prepared from uranyl nitrate hexahydrate (UO_2_(NO_3_)_2_·6H_2_O, Sigma–Aldrich, St. Louis, MO, USA) by dissolving its appropriate amounts. The water used was deionized by a water purification system.

### 3.2. Synthesis of ZIF-67 Nanoparticles

ZIF-67 nanoparticles were prepared through a one-step solution precipitation route at room temperature [26]. Specially, 20 mmol CoSO_4_∙7H_2_O and 160 mmol MeimiH (Figure S7) were dissolved in 400 mL methanol, respectively. Then, the methanol solution containing 160 mmol MeimiH was added into a methanol solution of CoSO_4_. Subsequently, the mixed solution was stirred for another 4 h. Finally, the obtained purple products were collected by filtration, washed with methanol for a few times and then dried for 12 h in a freezing drier at −60 °C.

### 3.3. Preparation of ZIF-67-PU

Firstly, the sponge with a size of 1 cm × 1 cm × 1 cm was cleaned several times with ethanol and deionized water, and dried in a vacuum desiccator at 60 °C for 24 h. Then, the dried sponges were clipped onto a baker containing 100 mL PAM solution with a concentration of 1 g∙L^−1^. The baker containing both sponges and PAM solution underwent ultrasonic treatment for 1 h to achieve PAM sedimentation on the sponge surface. Afterwards, the ZIF-67 sample with 100 mg was added into deionized water (100 mL) to prepare the ZIF-67 solution, which underwent ultrasonic processing for 30 min to achieve its uniform dispersion in deionized water. Next, the PAM-modified sponge was put into the homogeneous ZIF-67 solution, which underwent ultrasonic processing for another 3 h to realize the self-assembly of ZIF-67 and the PAM-modified sponge. Finally, the obtained ZIF-67-PU was cleaned several times with ethanol and deionized water to remove the redundant PAM molecules and dried at 60 °C.

### 3.4. Characterization

The obtained materials were characterized using XRD, FT-IR, SEM, EDS, and N_2_-adsorption–desorption measurements. The XRD (X-ray diffraction) patterns were collected using a Philips X’Pert Pro Super diffractometer (Amsterdam, The Netherland) at a scan rate of 2°/min using Cu-Kα radiation (λ = 1.54178 Å). The FT-IR spectrum of ZIF-67 was recorded (KBr disk, 400–4000 cm^−1^) by using a Nicolet-5700 FT-IR spectrophotometer (Thermoelectric Corporation of America, Madison, WI, USA). The SEM images and components of the prepared samples were conducted by using scanning electron microscopy (JSM-6700F, JEOL, Tokyo, Japan) and attached X-ray energy dispersive spectrometry (EDS), respectively. Before SEM measurement, the samples were attached to carbon tape and sputtered with a thin gold film to minimize the charging effects. N_2_ adsorption–desorption isotherms were obtained at liquid N_2_ temperature (77 K) using Quantachrome Autosorb IQ-C nitrogen adsorption apparatus (McMurdoch Co., Ltd., Norcross, GA, USA). Prior to adsorption, the samples were outgassed under a high vacuum for 16 h at 100 °C. A Shimadzu UV-3600 spectrophotometer (Shimadzu Co., Ltd, Tokyo, Japan) was used to estimate the U(VI) concentration using chlorophosphonazo III (chromogenic agent), by monitoring the absorption of the complex @ 669 nm. The chemical composition and chemical state of the material surface were analyzed by X-ray photoelectron spectroscopy through Themo Scientific K-Alpha X-ray photoelectron spectrometer (Thermo Fisher Scientific, Waltham, USA) using an EX06 ion source. The Zeta potential values were obtained using a Zetasizer (Nano-ZS) from Malvern Instruments (Malvern Co., Ltd., Worcestershire, UK).

### 3.5. Adsorption Experiment

The U(VI) elimination onto ZIF-67-PU was examined in aqueous solution through a batch method in particular environment conditions, containing the shaking time, pH value, temperature and initial U(VI) concentration. The acidity and alkalinity of the solution were regulated by using a negligible volume of dilute HNO_3_ or NaOH solution. And the adsorbent concentration applied in all batch tests remained constant at 0.5 g∙L^−1^.

The elimination amount of U(VI), *q_e_* (mg·g^−1^) was evaluated using the following equation:

$$q_{e}=\frac{(C_{0}-C_{e})V}{m}$$

where *C*_0_ (mg·L^−1^) is the initial concentration of U(VI), *C_e_* (mg·L^−1^) is the equilibrium concentration of U(VI), *m* (g) is the mass of the absorbent, and *V* (L) is the volume of the testing solution. All values were measured three parallel times to improve the reliability of the experiments.

## 4. Conclusions

In conclusion, self-assembled ZIF-67-PU was successfully fabricated by an in situ hybridization technique due to its simplicity and potential for mass-production. The ZIF-67-PU possessed a 3D porous skeleton. The resultant ZIF-67-PU exhibited better U(VI) elimination performance in comparison to pristine sponges and other adsorbents, which can be ascribed to the large BET surface area of ZIF-67 nanoparticles with its specific hollow sphere structure. In addition, U(VI) elimination onto ZIF-67-PU was realized through the inner-sphere complexation between U(VI) and uncoordinated functional groups C–N(H) (–OH) on the ZIF-67 surface, based on XPS investigations. This ZIF-67-PU represents a simple, feasible and low-cost disposal option for preparing ZIF-coated sponges of any shape to enhance the U(VI) elimination capacity. Furthermore, this in situ hybridization technique can be also extended to prepare other MOF-sponges through this self-assembled method.

## Figures and Tables

**Table 1:** *S_BET_*, *V_pore_* and average pore diameters of ZIF-67, PU and ZIF-67-PU.

Sample	*S_BET_* (m^2^·g^−1^)	*V_pore_* (cm^3^·g^−1^)	Pore Diameters (nm)
ZIF-67	1190.0	0.13	2.74
PU	1.0	0.02	1.95
ZIF-67-PU	3.0	0.01	1.67

**Table 2:** Parameters for pseudo-first-order and pseudo-second-order kinetic models.

Samples	Models	Pseudo-First-Order		Pseudo-Second-Order	
ZIF-67	Parameters	*k*_1_ (min^−1^)	0.1977	*k*_2_ (g·mg·min^−1^)	0.0186
		*q_e_* (mg·g^−1^)	65.30	*q_e_* (mg·g^−1^)	65.53
		*R* ^2^	0.9985	*R* ^2^	0.9998
PU	Parameters	*k*_1_ (min^−1^)	0.0805	*k*_2_ (g·mg·min^−1^)	0.0058
		*q_e_* (mg·g^−1^)	29.51	*q_e_* (mg·g^−1^)	30.30
		*R* ^2^	0.9874	*R* ^2^	0.9998
ZIF-67-PU	Parameters	*k*_1_ (min^−1^)	0.1324	*k*_2_ (g·mg·min^−1^)	0.0081
		*q_e_* (mg·g^−1^)	60.92	*q_e_* (mg·g^−1^)	61.20
		*R* ^2^	0.9932	*R* ^2^	0.9995

**Table 3:** Isotherms parameters for Langmuir, Freundlich and D-R models of the obtained materials.

Models	Adsorbent	Parameters		
Langmuir		*Q_0_* (mg·g^−1^)	*b* (L·mg^−1^)	*R* ^2^
	ZIF-67	389.23	0.7154	0.9811
	ZIF-67-PU	150.86	0.2719	0.9988
	PU	45.16	0.1085	0.9994
Freundlich		*K_F_* (mg^1−n^∙L^n^∙g^−1^)	*n*	*R* ^2^
	ZIF-67	183.03	1.223	0.9828
	ZIF-67-PU	34.94	1.701	0.9912
	PU	10.52	2.812	0.9872
D-R		*β* (mol^2^·J^−2^)	*E* (kJ·mol^−1^)	*R* ^2^
	ZIF-67	4.856 × 10^−9^	10.15	0.9822
	ZIF-67-PU	4.143 × 10^−9^	10.98	0.9983
	PU	2.996 × 10^−9^	12.92	0.9910

**Table 4:** Comparison of elimination capacity (mg·g^−1^) for U(VI) on ZIF-sponges with other absorbents.

Adsorbent Sample	Adsorption Capacity (mg·g^−1^)	pH	Reference
Biochar	2.12	3.9	[43]
Silicate/diatomite	31.54	5	[44]
Sulfonated GO	45.05	5	[45]
ZIF-90	77.6	4	[46]
ZIF-90-SA	85.1	4	[46]
Zn(HBTC)(L)·(H_2_O)_2_	115.0	2.0	[47]
UiO-68	217	2.0	[48]
MOF-76	298	3.0	[49]
MOF-3	304	7.0	[50]
FJSM-SnS	338.43	5.8	[51]
Fe-ZIF-8-300	343.5	4.5	[20]
BZCA-ZIF-8	429.56	3.0	[52]
HAP/ZIF-67	453.1	5.0	[22]
Fe_3_O_4_@ZIF-8	523.5	3.0	[53]
PAN@ZIF-8	530.3	3.0	[54]
CAU-1	648.37	4.0	[55]
Fe-ZIF-8-300-PO4	691.8	4.5	[20]
ZIF-67	389.23	5.5	This work
ZIF-67-PU	150.86	5.5	

## Data Availability

The data presented in this research are available upon request from the corresponding author.

## Supplementary Materials

The following supporting information can be downloaded at: https://www.mdpi.com/article/10.3390/molecules29020408/s1, Figure S1: XRD patterns of the obtained ZIF-67 material; Figure S2: (a) SEM image of the ZIF-67, (b) Corresponding EDS examination, (c–f) Elemental mapping images of ZIF-67; Figure S3: (a) SEM image of the ZIF-67-PU, (b) Corresponding EDS examination of ZIF-67-PU. Figure S4: (a) N_2_ adsorption–desorption isotherms of PU and (b) pore size distribution plot of PU calculated using the BJH method; Figure S5: (a,c) N_2_ adsorption–desorption isotherms of ZIF-67 nanoparticles and ZIF-67-PU after U(VI) adsorption, respectively and (b,d) pore size distribution plots of ZIF-67 nanoparticles and ZIF-67-PU after U(VI) adsorption, respectively, calculated using the BJH method; Figure S6: Distribution of aqueous U(VI) species as a function of the pH values; Figure S7: Zeta potential of the obtained MOF materials; Figure S8: Molecular structure of MeimiH. Table S1: Equations and nomenclatures of these kinetic models (*q_e_* and *q_t_* refer to the amounts of U(VI) adsorbed at equilibrium and designed time t, respectively); Table S2: The percent content of U(VI) species under different pH values; Table S3: The concentration of Co released during the adsorption process with an initial adsorbent concentration of m/V = 0.5 g·L^−1^.
