# Supplementary material for: Zeolitic Imidazole Framework (ZIF)–Sponge Composite for Highly Efficient U(VI) Elimination
Source: Molecules. 2024 Jan 15;29(2):408. doi: 10.3390/molecules29020408 (PMC10818524; doi:10.3390/molecules29020408)
Supplement: Supplementary file 1 [file molecules-29-00408-s001.zip › molecules-2759614-supplementary.pdf]

Supplementary Material

Zeolitic Imidazole Framework (ZIF)–Sponge Composite for Highly Efficient U(VI) Elimination

Shengxia Duan [1,2], Xinshu Long [1], Jian Liu [3,*], Xiaomin Jin [1], Guihong Zhao [3,*], Jiaxing Li [2] and Zaidao Liu [4]

1 Department of Chemistry and Engineering, Heze University, Heze 274500, China;
duanshengxia@hezeu.edu.cn (S.D.); longxinshu@sina.com (X.L.); 17661369763@163.com (X.J.)
2 CAS Key Laboratory of Photovoltaic and Energy Conservation Materials, Institute of Plasma Physics,
Chinese Academy of Sciences, Hefei 230031, China; lijx@ipp.ac.cn
3 College of Agriculture and Bioengineering, Heze University, Heze 274000, China
4 China National Nuclear Corporation Shaoguan JinYuan Uranium Co., Ltd., Shaoguan 512000, China; f15964152031@163.com
* Correspondence: liujianscholar@163.com (J.L.); sdzgh100@163.com (G.Z.)

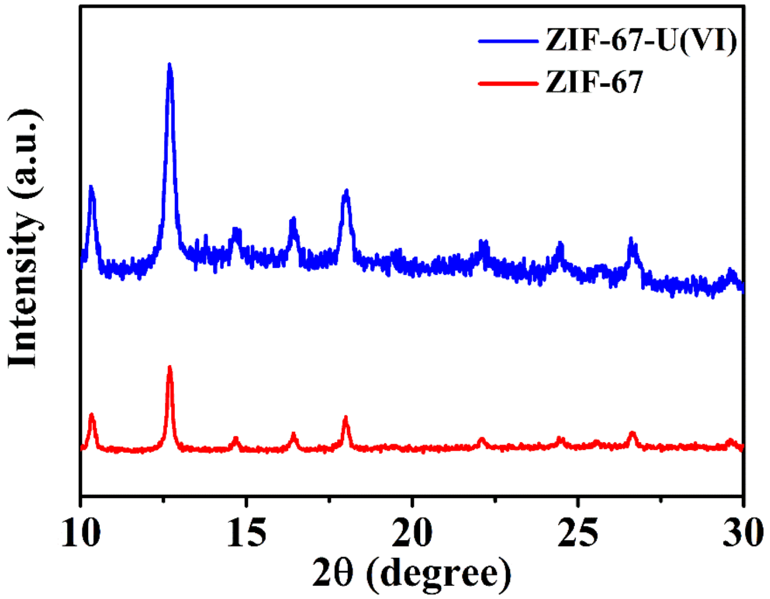

Figure S1 XRD patterns of obtained ZIF-67 material.

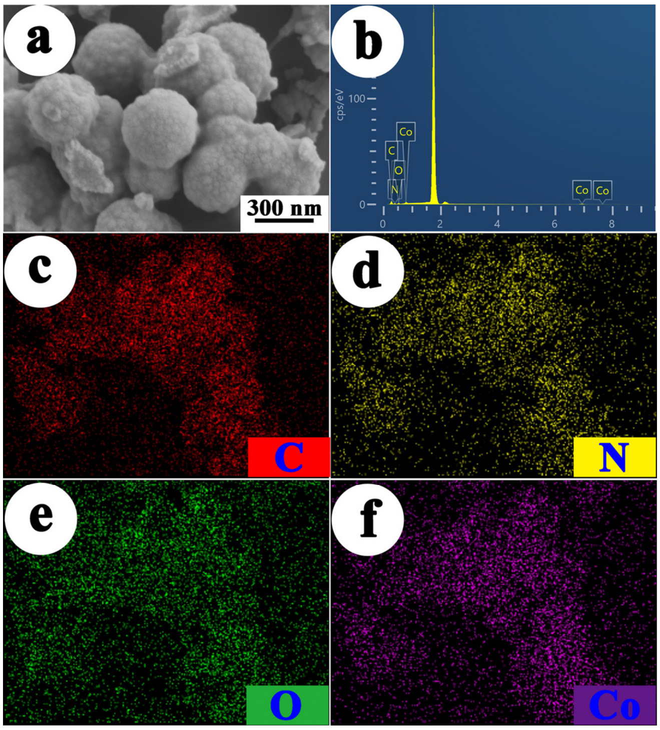

Figure S2 (a) SEM image of the ZIF-67, (b) Corresponding EDS examination, (c–f) Elemental mapping images of ZIF-67.

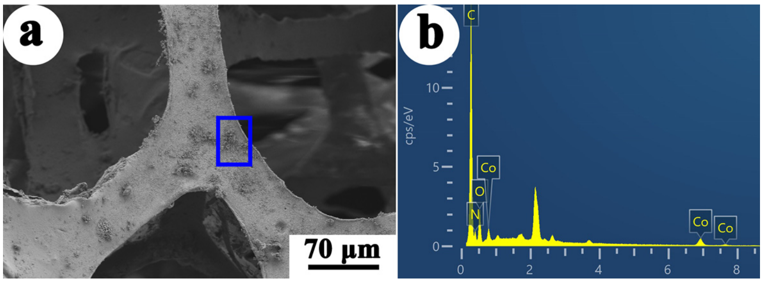

Figure S3 (a) SEM image of the ZIF-67-PU, (b) Corresponding EDS examination of ZIF-67-PU.

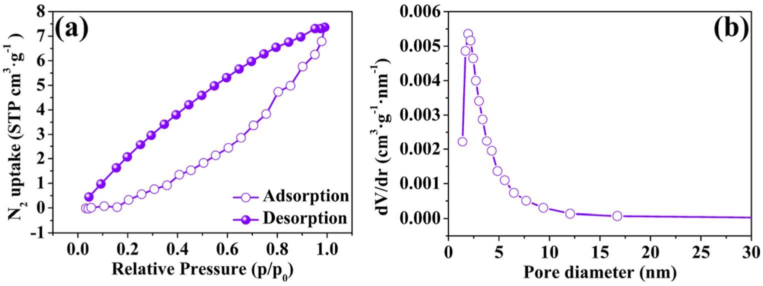

Figure S4 (a) N_2 adsorption–desorption isotherms PU, (b) pore size distribution plot of PU calculated using the BJH method.

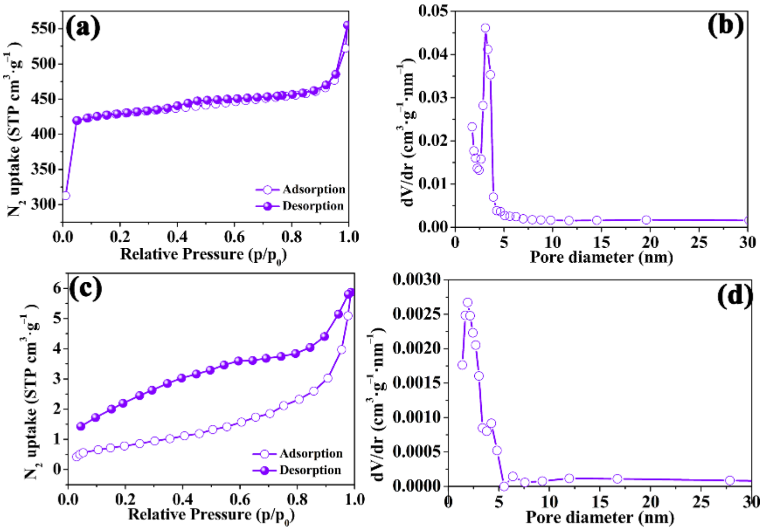

Figure S5 (a) and (c) N_2 adsorption–desorption isotherms of ZIF-67 nanoparticles and ZIF-67-PU after U(VI) adsorption, respectively, (b) and (d) pore size distribution plots of ZIF-67 nanoparticles and ZIF-67-PU after U(VI) adsorption, respectively, calculated using the BJH method.

Table S1 Equations and nomenclatures of these kinetic models (q_e and q_t refer to the amounts of U(VI) adsorbed at equilibrium and designed time t, respectively).

Equations	nomenclatures	
$q_t = q_e(1-e^{-k_1 t})$	k_1 (min^{-1})	pseudo-first order rate constant
$\frac{t}{q_t} = \frac{1}{2k_2 q_e^2} + \frac{1}{q_e}t$	k_2 (g·mg^{-1}·min^{-1})	pseudo-second order rate constant
$q_t = k_i t^{1/2} + C$	k_i (mg·g^{-1}·min$^{1/2}$)	intra-particle diffusion constant
	C	intercept in the intra-particle diffusion model

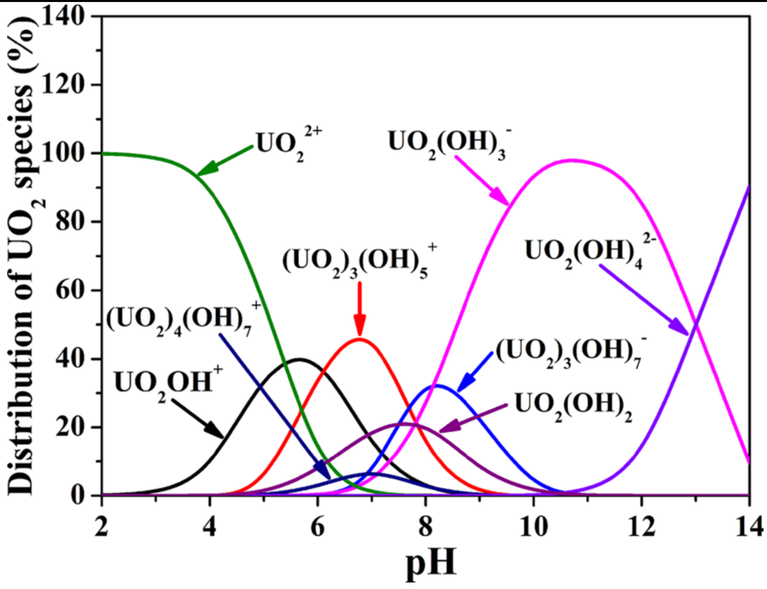

Figure S6 Distribution of aqueous U(VI) species as a function of the pH values.

Table S2 The percent content of U(VI) species under different pH values.

Species	pH=3	pH=4	pH=5	pH=6	pH=7
UO_2^{2+}	99.37%	93.99%	58.97%	7.0%	0.2%
UO_2OH^+	0.63%	37.20%	44.15%	36.64%	12.66%
$(UO_2)_3(OH)_5^+$			0.47%	36.64%	54.50%
$(UO_2)_4(OH)_7^+$				2.47%	8.37%
$UO_2(OH)_2$			0.59%	6.70%	20.06%
$(UO_2)_3(OH)_7^-$					2.07%
$UO_2(OH)_3^-$				0.07%	2.00%
Species	pH=8	pH=9	pH=10	pH=11	pH=12
UO_2^{2+}					
UO_2OH^+	1.44%	0.044%			
$(UO_2)_3(OH)_5^+$	10.59	0.061%			
$(UO_2)_4(OH)_7^+$	2.13%				
$UO_2(OH)_2$	22.81%	6.98%	0.98%		
$(UO_2)_3(OH)_7^-$	40.23%	23.77%	1.27%		
$UO_2(OH)_3^-$	22.80%	69.82%	97.65%	98.90%	90.91%

Table S3 The concentration of Co released during the adsorption process with initial adsorbent concentration of m/V = 0.5 g·L^{-1}.

$C_{o(U(VI))}$ (mg·L^{-1})	C_{co} (g/L)/ZIF-67	C_{co} (m·L^{-1})/ZIF-67-PU

21	0.46×10^{-2}	0.41×10^{-2}
27	0.53×10^{-2}	0.47×10^{-2}
33	0.56×10^{-2}	0.53×10^{-2}
39	0.78×10^{-2}	0.65×10^{-2}
45	0.87×10^{-2}	0.70×10^{-2}

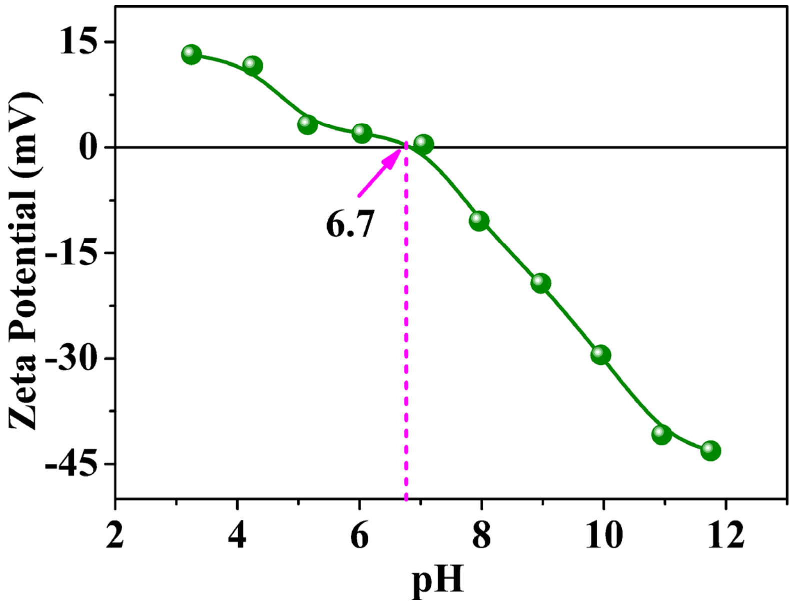

Figure S7 Zeta potential of the obtained MOFs materials.

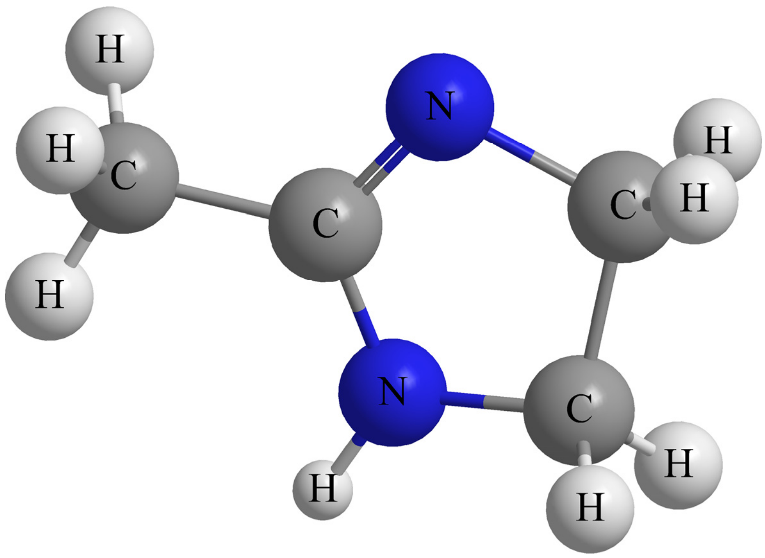

Figure S8 Molecular structure of MeimiH.
